# A preliminary survey of medium and large‐sized mammals from Lebu Natural Protected Forest, Southwest Showa, Ethiopia

**DOI:** 10.1002/ece3.5733

**Published:** 2019-10-22

**Authors:** Chala Adugna Qufa, Afework Bekele

**Affiliations:** ^1^ Department of Biology Woldia University Woldia Ethiopia; ^2^ Department of Zoological Sciences Addis Ababa University Addis Ababa Ethiopia

**Keywords:** diversity, ethiopia, Lebu forest, mammal survey

## Abstract

This study was conducted to determine the species composition and diversity of medium and large‐sized mammals from Lebu Natural Protected Forest, Ethiopia. Surveys were conducted to record mammals through direct observation and indirect evidence from three habitat types, namely: natural forest, bushland, and riverine forest. A total of 15 mammalian species were recorded. The species recorded were *Papio anubis*, *Chlorocebus aethiops*, *Tragelaphus scriptus*, *Canis aureus*, *Crocuta crocuta*, *Panthera pardus*, *Procavia capensis*, *Colobus guereza*, *Sylvicapra grimmia*, *Orycteropus afer*, *Helogale parvula*, *Hystrix cristata*, *Lepus fagani*, *Potamochoerus larvatus*, and *Phacochoeus africanus*. A total of 223 records of observations were compiled. About 74% of these records (*N* = 167) were obtained from direct sight, whereas the rest was recorded through indirect evidence. The dominant order recorded was order Primates (57.4%) followed by order Artiodactyla (17.5%) while the least record was order Lagomorpha (1.34%). The species richness varied across the stratified habitat types. However, there is no significant difference in Shannon–Wiener Index values between the habitat types. The species diversity of the study area was *H*′ = 2.119. The present study area is of great potential area for the conservation of the species. Long‐term detailed studies should be carried out for effective conservation and management initiatives in the study area.

## Graphical abstract

Medium‐ and large‐sized mammals from Lebu Natural Protected Forest of Ethiopia were surveyed. The area will contribute to the conservation of the mammal species. Long‐term detailed studies should be carried out for effective conservation and management initiative in the study area.

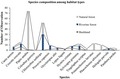

## 1 INTRODUCTION

Mammalian species are one of the greatest resources found on the earth. Mammals are the most important components of terrestrial ecosystems (Bogonia et al., 2017) and provide vital ecological functions such as pollination (Mora, Méndez, & Gómez, 1999), seed dispersal (Alves‐Costa & Eterovick, 2007; Botelho, Calouro, Borges, & Chaves, 2012), and predation (Botelho et al., 2012; Weckel, Giuliano, & Silver, 2006). They are also vital constituents of ecosystems (Boddicker, Rodriguez, & Amanzo, 2002), keeping ecological stability (Herrerias‐Diego et al., 2008). They are considered as an important resource for humankind and provide benefits such as a source of food and income generation like tusks, horns, and ivory (Boesch, Mundry, Kühl, & Berger, 2017).

Mammals are threatened by various factors induced by human beings. Landscape modification and habitat fragmentation are major factors for species loss (Fischer & Lindenmayer, 2007; Johnstone, Reina, & Lill, 2010; Prugha, Hodgesb, Sinclairc, & Brasharesa, 2008). Expansion in agricultural schemes, deforestation, desertification, and hunting, resulting from economic activities which may cause loss to mammals (Bernardo & Melo, 2013; Kasso & Bekele, 2014; Wale, Kassie, Mulualem, Tesfahunegny, & Assefa, 2017). To overcome such enormous pressure from the mammals, conserving and managing them in and outside protected areas is a must among the nations of the world.

Mammals are the most diverse and successful group of animals having approximately 5,416 extant species on the globe (Geleta & Bekele, 2016; Reale, Fonseca, & Uieda, 2014). About 320 species of mammals exist in Ethiopia of which 55 are endemic (Lavrenchenko & Bekele, 2017). Ethiopia possesses wide geographic, topographic, and climatic variations (Tefera, 2011). The variety of conditions created in a given ecosystem that harbors diversified‐habitats that serves as home to a large number of endemic mammal species (Bantihun & Bekele, 2015; Yalden & Largen, 1992). A basic requirement for determining the status of species is surveying mammals (Keeping & Pelletier, 2014). Mammal inventories are essential tools to efficiently forward conservation strategies and management practices (Legese, Bekele, & Kiros, 2019). There are several previous studies conducted in Ethiopia based on mammals. Yalden (1988) investigated small mammals of Bale Mountains National Park; Yalden and Largen (1992) reviewed the endemic mammals of Ethiopia and Yalden, Largen, Kock, and Hillman (1996) identified Ethiopian and Eritrean mammal fauna in their review and recognize provisional totals of 277 terrestrial and 11 marine species. Woldegeorgis and Wube (2012) surveyed mammals of the Yayu forest in the southwest Ethiopia; Kasso and Bekele (2014) investigated threats to mammals on fragmented habitats around Asella Town, Central Ethiopia and Geleta and Bekele (2016) surveyed medium and large‐sized mammals in Wacha Protected Forest, Western Ethiopia. Wale et al. (2017) also assessed the threats to wildlife and its relative severity from Eastern Ethiopia Protected Areas; Kasso and Bekele (2017) assessed the diversity, abundance and distribution of mammals in fragmented remnant forests around Asella Town, Ethiopia and Atnafu and Yihune (2018) investigated the species composition and relative abundance of medium and large mammals in Mengaza communal forest, East Gojjam, Ethiopia. However, still there is a need to conduct studies in fragmented forest of the country.

Even though studies conducted on mammals, mainly targeted National Parks and sanctuaries (Kasso & Bekele, 2014), the survey on fragmented forest and scrubs is scanty. Knowledge regarding the conservation status of Ethiopian mammals is fragmentary (Saavedra et al., 2009) as a vast area remains biologically unexplored due to a major habitat block within the country. A complete inventory of mammals on different ecosystem types of Ethiopia does not exist and is not well documented (Tefera, 2011). Documents on the status and trends of mammals in various protected areas are needed if they are to be protected and managed. Among various fragmented protected forests in Ethiopia, Lebu Natural Protected Forest is one of them, believed to harbor different mammalian species. The main objective of the present study was to determine the species composition, diversity and relative abundance of medium and large‐sized mammals from Lebu Natural Protected Forest, Southwest Showa, central Ethiopia.

## 2 MATERIALS AND METHODS

### 2.1 Study area description

**Figure 1:**
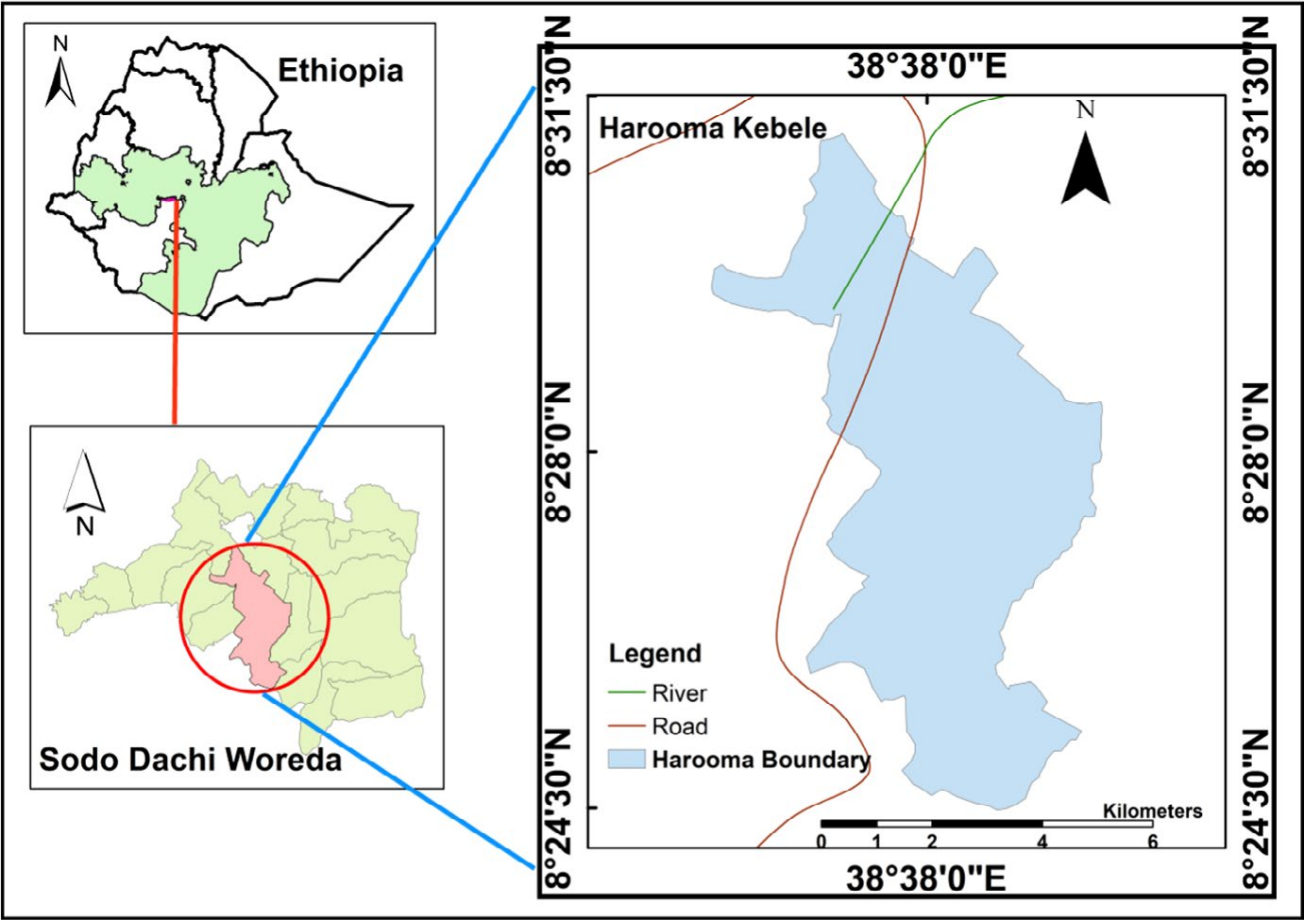
Map of Sodo Dachi Woreda, Harooma Kebele, Oromia Regional State, Ethiopia

Lebu Natural Protected Forest (between 8°28′42″N and 38°39′24″E) is found in the Southwest Shoa Zone, Oromia Regional State, Ethiopia. It is located in Sodo Dachi Woreda; about 110 km away from Addis Ababa, the capital city of Ethiopia (Figure 1). The total area of the forest is around 32 hectares bounded by Gara Molcha Kebele to the north, Kerchufa Kebele to the east, Suten and Tiya Town to the south, and Cheeka Kebele to the west. The area was designated by the Wordea's Natural Resource Department as a protected area.

A climatic data for the study area of ten consecutive years were obtained (ENMA, 2018). The study area has unimodal rainfall distribution, a long rainy season from June to September and a dry season from November to February. The average mean monthly maximum temperature is 26.85°C, and the average mean monthly minimum temperature is 12.05°C. The mean monthly rainfall of the area varies between 3.5 mm (December) and 346.0 mm (July), while the average mean monthly rainfall of the area is 139.08 mm.

The study area was stratified into three habitat types based on the types of vegetation structure and land cover. These habitats, include: natural forest, bushland, and riverine forest. The dominant or abundant plant species identification was made in situ based on the researchers' previous knowledge of the plant vernacular name and a field guide book. Then, within these strata plant specimens were randomly sampled, observed, identified into taxonomical group. Besides, a field guide book of useful trees of Ethiopia and the Flora of Ethiopia and Eritrea, written by Bekele (2007) and Phulips (1995), was used to compare the morphological features of plant found in their natural habitat. Accordingly, across the entire study area plant growth forms such as trees, shrubs, and herbs were identified. The identified trees from Lebu Natural Protected Forest were included; *Acacia sieberiana*, *Acokanthera schimperi*, *Calpurnia aurea*, *Cordia Africana*, *Croton macrostachyus*, *Eucalyptus camaldulensis*, *Euphorbia abyssinica*, *Ficus vasta*, *Juniperus procera*, and *Olea europaea*. Shrubs recorded were *Bersama abyssinica*, *Capparis tomentosa*, *Carissa spinarum*, *Dodonaea angustifolia*, *Euclea divinorum*, *Lippia adoensis*, *Myrsine africana*, *Osyris quadripartita*, *Premna schimperi*, *Pterolobium stellatum*, *Rhamnus staddo*, *Rhus vulgaris Meikles*, and *Rubus steudneri*. *Bidens pilosa*, *Crassocephalum macropappum*, *Cyathula uncinulata*, *Hyparrhenia dregeana*, and *Solanum incanum* were the major dominant herbs found in the present study area.

### 2.2 Methods of data collection

For this study, the study area was stratified into three patches of habitats based on the vegetation structures and topography of the landscapes. These habitats, include: natural forest (6 km^2^), bushland (2 km^2^), and riverine forest (8 km along). Diurnal line‐transect is a well‐recognized and cost‐effective methodology for surveying medium and large vertebrates in tropical forests and savannas (Effiom, Nuñez‐Iturri, Smith, Ottosson, & Olsson, 2013; Haugaasen & Peres, 2005; Ogutu, Bhola, Piepho, & Reid, 2006). It is one of the best methods for estimating abundance of relatively large and conspicuous mammals (Krebs, 2006). So, combining diurnal line‐transect with indirect surveys (including fresh tracks, feces, hair, horns, burrows and digging) can enhance the detectability for many mammal species, contributing to maximize the species lists (Larsen, 2016).

A survey was conducted for seven consecutive days when the activities of mammals were more active: early morning from 6:00 to 10:00 and from 16:00 to 19:00 (Legese et al., 2019). During data collection, the observers were walking on foot along the line transect established and directly count all the individuals sighted with their respective species using unaided eyes and binocular. A total of 12 randomly laid transect line (four of each habitat type) were established to count the sighted mammals. Also, the researcher and two field assistants walk in a transect line and recorded indirect evidence of the animal signs in each sampled area and identified. Surveys were conducted during the dry season from October to December, 2018. Besides, information on lists of species, the altitude, coordinate systems, and vegetation features was recorded by Garmin 72 Geographic Positioning System when the accuracy level read below 10. The distribution of mammals found in Lebu Natural Protected Forest was based on the presence or absence of the species in the habitats categorized. Finally, rarefaction curve can be computed to compare the mean of species richness among the three habitat types from the study area (Colwell, Mao, & Chang, 2004).

### 2.3 Data analysis

Data were analyzed using descriptive statistics and species diversity index. Mammalian species diversity of the study area was computed using Shannon–Weaver Index of diversity: *H*′ = −∑ *Pi*ln*Pi*, where *H*′ is the Shannon index of diversity, *Pi* is the proportion of individuals of a species in a sample and ln = Natural logarithm (Shannon & Weaver, 1949). Simpson index of diversity was followed (1 − *D*) using the formula: *J* = *H*′/*H*′ max, where *H*′ is the observed index of diversity and *H*′ max = ln(*S*); *S* = the number of species in each habitat; ln = Natural logarithm was computed to determine the evenness and dominance among the mammalian species. Finally, relative abundance was used to compute for each species occurrence in the study area.

## 3 RESULTS

The study compiled 223 observational records, belonging to 15 species and grouped into seven orders and 11 families (Table 1). Among the seven orders identified, order Carnivora and order Artiodctayla were represented by four species each and the other orders have one species except order Primates represented by three species. Based on trophic guilds, mammal species recorded such as *Canis aureus*, *Crocuta crocuta*, *Panthera pardus*, and *Helogale parvula* were carnivores and the remaining majority were herbivores. The majority of the recorded mammal species has terrestrial habits. The number of individual observations recorded and there the relative frequency of each mammal species was presented in Table 2. *Papio anubis* has the highest relative frequency of 33.12% (*N* = 74), and the least relative frequency of 0.8% (*N* = 2) was for *Coloubus guereza*.

**Table 1:** List of species, common name, local name, habitat type, and type of record of mammals identified from Lebu Natural Protected Forest

Scientific name	Common name	Habitat type	Mode of record
Cercopithecidae			
*Papio anubis*	Olive baboon	RF, NF	SC, FP
*Chlorocebus aethiops*	Verevet monkey	RF, NF	SC
*Coloubus guereza*	Coloubus monkey	NF	SC
Bovidae			
*Sylvicapra grimmia*	Bush duiker	BL, RF, NF	SC, TC
*Tragelaphus scriptus*	Bushbuck	NF	SC, TC
Suidae			
*Potamochoerus larvatus*	Bush pig	NF	SC, FP
*Phacochoeus africanus*	Common warthog	NF	SC, FP
Canidae			
*Canis aureus*	Common jackal	BL, RF, NF	FP
Hyaenidae			
*Crocuta crocuta*	Spotted hyaena	BL, RF, NF	FP, TC
Felidae			
*Panthera pardus*	Leopard*	NF	–
Herpestidae			
*Helogale parvula*	Common mongoose	BL, RF	FP
Procaviidae			–
*Procavia capensis*	Rock hyrax	BL, RF	SC
Orycteropodidae			
*Orycteropus afer*	Aardvark	BL, RF	BH
Leporidae			
*Lepus fagani*	Bush hare	BL	SC
Hysticide			
*Hystrix cristata*	Porcupine	BL, RF	FP, BH, TC

Abbreviations: BH, burrow/horns counts; BL, bushland; DS, direct sight; NF, natural forest; RF, riverine forest; SC, scat counts; TC, track counts.

* Villager's assurances of presence.

**Table 2:** Mammal species record and their relative frequency observations during the survey period

Order	Family	Common name	Scientific name	No. of observation	Relative frequency observation
Carnivora	Canidae	Common jackal	*Canis aureus*	8	3.59
	Hyaenidae	Spotted hyena	*Crocuta crocuta*	15	6.73
	Felidae	Leopard^a^	*Panthera pardus*	4	1.79
	Herpestidae	Common dwarf mongoose	*Helogale parvula*	4	1.79
Primates	Cercopithecidae	Olive baboon	*Papio anubis*	74	33.12
		Coloubus monkey	*Coloubus guereza*	2	0.89
		Vervet monkey	*Chlorocebus aethiops*	52	23.32
Hyracoidea	Procaviidae	Rock hyrax	*Procavia capensis*	9	4.04
Tabulidentata	Orycteropodidae	Aardvark	*Orycteropus afer*	5	2.24
Lagomorpha	Leporidae	Bush hare	*Lepus fagani*	3	1.34
Rodentia	Hysticide	African porcupines	*Hystrix cristata*	8	3.59
Artiodactyla	Bovidae	Common duiker	*Sylvicapra grimmia*	12	5.38
		Bushbuck	*Tragelaphus scriptus*	4	1.79
	Suidae	Bushpig	*Potamochoerus larvatus*	13	5.83
		Common warthog	*Phacochoeus africanus*	10	4.48

a Villager's assurances for presence.

*Crocuta crocuta* was with the highest frequency of 6.73% (*N* = 15) and *H. parvula* with the lowest frequency of 1.79% (*N* = 4). Orders Hyracoidea, Tubulidentata, Rodentia, and Lagomorpha were represented only by a single species. The dominant order by the number of observations from the study area was recorded by order Primates followed by order Artiodactyla. The most dominant species recorded from order Primates were olive baboon 33.12% (*N* = 74), and the dominant species recorded from order Artiodactyla was *Potamochoerus larvatus* 5.83% (*N* = 13).

**Figure 2:**
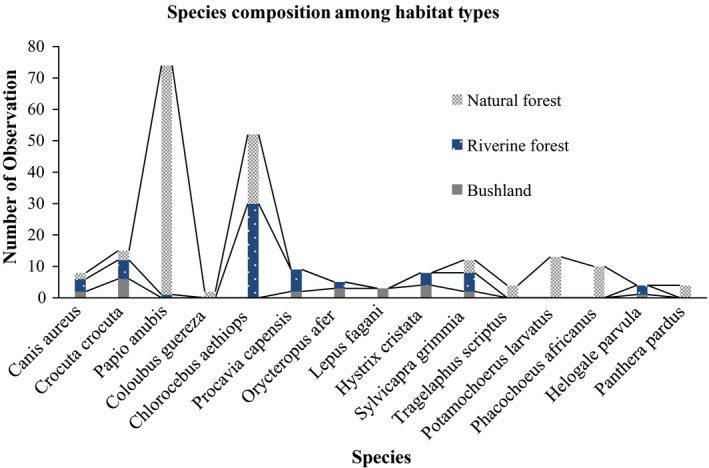
Number of species observed among the three stratified habitat types

The species richness varied across the habitat types stratified (Figure 2). About 15 species recorded, the species richness of the categorized habitats were 10, 9, and 8 for natural forest, riverine forest, and bushland, respectively. The total number of observations for mammals in the natural forest was *N* = 137, riverine forest *N* = 63, and bushland *N* = 23.

A total of 223 records of observations were compiled from Lebu Natural Protected Forest, and 74% of these records (*N* = 167) were obtained from direct sight followed by fecal‐pellet counts 13% (*N* = 30); animal pugmark counts 8% (*N* = 18) as well as burrow and horn counts 3.5% (*N* = 8) (Table 3). Nine of the recorded mammal species such as *P. anubis*, *Chlorocebus aethiops*, *Tragelaphus scriptus*, *Procavia capensis*, *Colobus guereza*, *Sylvicapra grimmia*, *Lepus fagani*, *Potamochoerus larvatus*, and *Phacochoeus africanus* were observed using direct sighting whereas the remaining records of observation for mammals from the study area were through indirect evidence. However, species *P. pardus* was assured of its presence from the local villagers.

**Table 3:** List of species, mode of identification and their respective observation in each habitat type

Species	Bushland				Riverine forest				Natural forest				Total
	SC	FP	TC	BH	SC	FP	TC	BH	SC	FP	TC	BH	
Common jackal	–	2	–	–	–	4	–	–	–	2	–	–	8
Spotted hyaena	–	3	3	–	–	3	3	–	–	3	–	–	15
Olive baboon	–	–	–	–	–	1	–	–	73	–	–	–	74
Coloubus monkey	–	–	–	–	–	–	–	–	2	–	–	–	2
Vervet monkey	–	–	–	–	30	–	–	–	22	–	–	–	52
Rock hyrax	2	–	–	–	7	–	–	–	–	–	–	–	9
Aardvark	–	–	–	3	–	–	–	2	–	–	–	–	5
Bush hare	3	–	–	–	–	–	–	–	–	–	–	–	3
African porcupines	–	1	1	2	–	–	3	1	–	–	–	–	8
Common duiker	–	–	2	–	1	–	5	–	4	–	–	–	12
Bushbuck	–	–	–	–	–	–	–	–	3	–	1	–	4
Bush pig	–	–	–	–	–	–	–	–	11	2	–	–	13
Common warthog	–	–	–	–	–	–	–	–	5	5	–	–	10
Mongoose	–	1	–	–	–	3	–	–	–	–	–	–	4
Leopard*	–	–	–	–	–	–	–	–	4	–	–	–	4
Total	5	7	6	5	38	11	11	3	124	12	1	–	223

Abbreviations: BH, burrow/horns count; FP, fecal‐pellet counts; SC, sighting counts; TC, track counts.

* Villager's assurances of presence.

**Figure 3:**
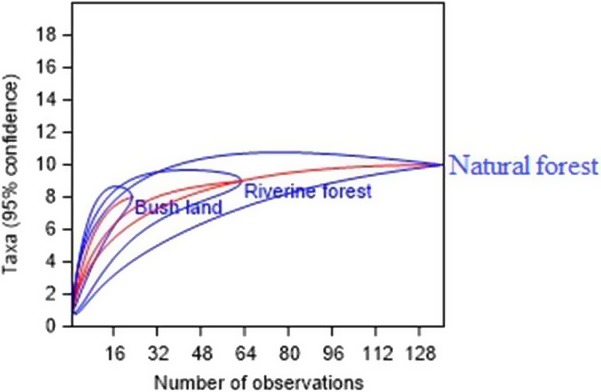
Mean number of species richness computed by rarefaction curve among the three stratified habitat types

The results of the present study showed that of the 223 total observations, 61.4% (*N* = 137) was recorded in the natural forest, 28.25% (*N* = 63) in the riverine forest and 10.3% (*N* = 23) in the bushland habitats (Figure 3). The diversity in abundance of records within and between the habitat types were given in Table 4. Also, the diversity of mammal species in each habitat type and of the study area were determined (Table 5). The highest species richness was recorded in the natural forest *N* = 10, and the least eight species were recorded in the bushland. The Shannon diversity of mammal species in the bushland was *H*′ = 1. A total of 96 higher than the remaining habitat types. But, there was no significant difference in Shannon–Wiener Index values between the three habitat types. The higher and lower evenness of the mammalian species was recorded in bushland (*E* = 0.887) and natural forest (*E* = 0.44). The dominance of mammalian species was recorded from the highest to the lowest in the natural forest (*D* = 0.3275) and bushland (*D* = 0.1569), respectively. The overall species richness of Lebu Natural Protected area was 15, and Shannon–Wiener Index values were low (*H*′ = 2.119) whereas the Simpson's index of diversity showed the highest species diversity (0.8167) in the study area.

**Table 4:** Diversity in abundance of records within and between habitat types

Species	Bushland	RA	Riverine forest	RA	Natural forest	RA	Overall record	RA
*Canis aureus*	2	8.70	4	6.35	2	1.46	8	3.59
*Crocuta crocuta*	6	26.09	6	9.52	3	2.19	15	6.73
*Papio anubis*	0	0.00	1	1.59	73	53.28	74	33.18
*Coloubus guereza*	0	0.00	0	0.00	2	1.46	2	0.90
*Chlorocebus aethiops*	0	0.00	30	47.62	22	16.06	52	23.32
*Procavia capensis*	2	8.70	7	11.11	0	0.00	9	4.04
*Orycteropus afer*	3	13.04	2	3.17	0	0.00	5	2.24
*Lepus fagani*	3	13.04	0	0.00	0	0.00	3	1.35
*Hystrix cristata*	4	17.39	4	6.35	0	0.00	8	3.59
*Sylvicapra grimmia*	2	8.70	6	9.52	4	2.92	12	5.38
*Tragelaphus scriptus*	0	0.00	0	0.00	4	2.92	4	1.79
*Potamochoerus larvatus*	0	0.00	0	0.00	13	9.49	13	5.83
*Phacochoeus africanus*	0	0.00	0	0.00	10	7.30	10	4.48
*Helogale parvula*	1	4.35	3	4.76	0	0.00	4	1.79
*Panthera pardus*	0	0.00	0	0.00	4	2.92	4	1.79
Total	23	100	63	100	137	100	223	100

**Table 5:** Species diversity indices between habitats during the survey period

Species diversity index	Bushland	Riverine forest	Natural forest	Overall diversity indices
Taxa_S	8	9	10	15
Individuals	23	63	137	223
Dominance_D	0.1569	0.2688	0.3275	0.1833
Simpson_1‐D	0.8431	0.7312	0.6725	0.8167
Shannon_H	1.96	1.716	1.56	2.119
Evenness_e^H/S	0.887	0.6178	0.476	0.555

## 4 DISCUSSION

During the present preliminary survey of large and medium‐sized mammals from Lebu Natural Protected Forest, a total of 15 species were identified from 223 total observational records. These mammal species were grouped into seven orders and eleven families. Similarly, Geleta and Bekele (2016) recorded 15 mammal species in Wacha Protected Forest, Western Ethiopia by direct and indirect evidences. Also, Woldegeorgis and Wube (2012) recorded 14 mammal species from Yayu forest in southwest Ethiopia. However, Atnafu and Yihune (2018) recorded lower (12) mammal species in the Mengaza communal forest, East Gojjam, Ethiopia. In contrast, research carried out in fragmented remnant forests around Asella town showed a total of 22 mammalian species (Kasso & Bekele, 2017). Tilahun and Merewa (2016) recorded a total of 19 species of large mammals in Tululujia Wildlife Reserve, southwestern Ethiopia. This variation might account for variation in mammal's group composition, variation in vegetation structure and human influence due to intensive deforestation, agricultural expansion, charcoal production, fuelwood sale, and expansion of rangelands for their livestock.

Studies conducted in different countries revealed that the medium and large‐sized mammals recorded were higher than the result obtained from the present study. Some of the studies among others include, Alves, Junior, and Brites (2014), Njoroge et al. (2009), Cortés‐Marcial, Ayón, and Briones‐Sala (2014), Brncic, Amarasekaran, McKenna, Mundry, and Kuhl (2015), Andrade Melo, Gadelha, Silva, Silva Júnior, and Pontes (2015), Campos, Lage, and Ribeiro (2013), Oliveira and Hannibal (2017), and Botelho et al. (2012) which recorded 18, 23, 18, 35, 33, 23, 22, and 27, respectively. Alves et al. (2014) recorded 18 in a fragment of Cerrado in the Triângulo Mineiro region, southeastern Brazil. This might account for variation in sample sites, sampling effort spent, season considered, and variation in vegetation physiognomy. Although due to the presence of top predators, Lebu Natural Protected Forest likely accommodates the less number of mammals than a fragment of Cerrado in the Triângulo Mineiro region, southeastern Brazil. Njoroge et al. (2009) recorded 23 species in Arawale National Reserve, Kenya, East Africa, which remains a stronghold for the endemic species that justify the conservation of the reserve. The present study area, however, was not given due attention to the biodiversity conservation and landscape restoration in the past decade. Cortés‐Marcial et al. (2014) recorded 18 species of large and medium‐sized mammals in Juchitan, Isthmus of Tehuantepec, Oaxaca, Mexico. This could create the area to preserve of large and medium‐sized mammals because the area is one of the country's most important regions from a zoogeographical perspective due to the large number of endemic Neotropical species found there. But, this is not the case in our study area. The number of species found in our study was dissimilar to that reported by Brncic et al. (2015) in the human‐dominated land‐use mosaic of Sierra Leone. However, their study was conducted throughout Sierra Leone to make inferences about species persistence and counted 35 large mammal species. Andrade Melo et al. (2015) recorded 33 mammal species; Campos et al. (2013) recorded 23 species mammals in Brazil; Oliveira and Hannibal (2017) recorded 22 species in fragmented, semi‐deciduous forest of Brazil and Botelho et al. (2012) recorded 27 species of large to medium‐sized mammals in Humaitá Forest Reserve, southwestern Amazonia, and State of Acre, Brazil. These variations might account for variations in climatic condition, census effort spent, variation in vegetation physiognomies, and other environmental characteristics.

The orders of the mammalian species recorded in the present study were inconsistent with the study conducted in Yayu forest by Woldegeorgis and Wube (2012) as seven orders of mammalian species were recorded here in Lebu Natural Protected Forest whereas only four of the orders namely: Primates, Artiodactyla, Carnivora, and Lagomorpha were observed there. This might account for agronomic practice in the buffer zone, such as some economic activities like collection of forest resources, including coffee that makes Yayu forest accommodating fewer wildlife species than Lebu Natural Protected Forest. The majority of the recorded mammal species has terrestrial habits while very few of them were arboreal. The proportion of large to medium‐sized mammals was more than half during the surveys in the study area. According to the conservation concern as per the IUCN (2016) Red List of Threatened species, the leopard, *P. pardus* global population is decreasing and is listed as a vulnerable species (Stein et al., 2016).

The dominant order in relative frequency record observation (relative abundance) was recorded by order Primates (57.4%) followed by order Artiodactyla (17.5%). From the primate groups, olive baboon has the highest relative frequency 33.12% (*N* = 74), the most dominant species in the area and *P. larvatus* having 5.83% (*N* = 13) was the dominant recorded from order Artiodactyla. These results were consistent with the study conducted in the Mengaza communal forest (Atnafu & Yihune, 2018). This similarity might be due to the availability of a variety of resources in both areas in which most herbivores depended on and particularly olive baboons are well adapted to feed on a variety of food items (Geleta & Bekele, 2016). From order Carnivora, the highest relative frequency was recorded for *C. crocuta* 6.73% (*N* = 15) and the least was for *H. parvula* 1.79% (*N* = 4). The low frequency of observation for carnivores might be due to their nocturnal habits, avoidance of their visualization as they are shy and the inaccessibility of the night survey in the study area. The majority of the species recorded in the present study area has diurnal habits in their activity patterns. This result was inconsistent with the result obtained by Alves et al. (2014) in which out of a total of 239 records, 75% (*N* = 178) were obtained from footprints.

Olive baboon (*P. anubis*) was the most abundant species, both in the natural forest habitat type and in the present study area. Similarly, Girma, Mamo, and Ersado (2012) confirmed that the most abundant species in and around Wondo Genet Forest Patch, Southern Ethiopia was *P. anubis*. This might be attributed to the behavior of the species known to be widely distributed in a variety of habitat from savanna grassland to Afro‐Montane forest. Besides, *P. anubis* was considered as generalists inhabiting different habitats (Mullu & Solomon, 2016). Also, this species might account to use the natural forest to escape from the local people attack to prevent their crops‐ride from wildlife.

The second most abundant in the study area and the highest abundant within the riverine forest was *C. aethiops*. Similarly, Legese et al. (2019) reported that this species was abundant in riverine forests. This might account for the presence of dense riparian vegetation which enables the species to shelter with and easily accesses the product of the trees such as fruits. Besides, Legese and co‐authors suggested that the presence of this species at the edge of riverine forest account with a system of feeding on crops from a shorter distance.

The species richness varied across the habitat types stratified. The mean species richness computed showed that the natural forest has the highest species richness than the rest two habitat types. Similar results were obtained by Geleta and Bekele (2016) as openness of the habitat which might have resulted from habitat loss and fragmentation leading to the species to be easily detected in the natural forest. In the present study, sparsely vegetated natural forest enhanced the detection of mammals. However, a limitation posed by the massive rocky gorge prevented us from making intensive searching throughout the entire course of the natural forest. This might be a reason for the low mammal species record in the area.

Species index of diversity showed that there is a variation in species diversity among the habitats. For instance, bushland forest has the highest species diversity (*H*′ = 1.96) while the least species diversity was recorded from the natural forest (*H*′ = 1.56). Similarly, studies conducted by Kasso and Bekele (2017) showed that variation in the number and abundance of mammal species among different habitats is related to the quality of the habitat and preference of the species. Our finding is inconsistent with the study conducted by Geleta and Bekele (2016), in which they obtained higher species diversity in the natural forest. These variations might be due to human pressure on Lebu Natural Protected Forest before it was protected. The overall species index of diversity of the study area showed minimal species richness (*H*′ = 2.119). However, there is no variation between the habitat types and number of mammalian species recorded. Different possible factors contributed to this. It might be due to lower survey period and habitat fragmentation.

## 5 CONCLUSIONS

The present study area has a representative species of medium and large‐sized mammals. Notable previous history of human influence such as intensive deforestation for agricultural expansion, charcoal production, fuelwood collection, and grazing by livestock resulted in alteration of the natural forest in the study area. This finding showed that attention should be given to the varieties of mammal species to avoid any aspect of human pressures on the protected forest. Therefore, joint conservation practice with the local community should be initiated to conserve and enhance the welfare of mammals that occur in the area. In so doing, the entrance of people and their livestock for grazing in the natural forest must be prevented. Long‐term comprehensive assessment of mammals needs to be documented and provision of knowledge‐based conservation and management initiatives must be given in the area.

## CONFLICT OF INTEREST

The authors declare that there is no competing interests in conflicts.

## AUTHOR CONTRIBUTIONS

Chala Adugna Qufa and Afework Bekele conceived, designed the study data collection. Chala Qufa conducted fieldwork, analysis, write the manuscript and revised the whole document. Afework Bekele designed the survey method, edit the manuscript and revised the final version of the main document for submission for potential review. All authors contributed to the writing of the manuscript and approved the submitted version.

## Data Availability

All data used in this study are archived in the Dryad data repository (Available here: DOI: https://doi.org/10.5061/dryad.g5gk675).
